# Purification and characterization of a novel glutamate dehydrogenase from *Geotrichum candidum* with higher alcohol and amino acid activity

**DOI:** 10.1186/s13568-016-0307-8

**Published:** 2017-01-03

**Authors:** Jing Zhu, Kuan Lu, Xiaoguang Xu, Xinglong Wang, Junling Shi

**Affiliations:** 1 Key Laboratory for Space Bioscience and Biotechnology, School of Life Sciences, Northwestern Polytechnical University, 127 Youyi West Road, Xi’an, 710072 Shaanxi China; 2 School of Food Science, Xinyang Agriculture and Forestry University, New 24 street of yangshan new district, Xinyang, 464000 Henan China; 3 College of Enology, Northwest A&F University, 23 Xinong Road, Yangling, 712100 Shaanxi China

**Keywords:** Hexanol, Glutamate dehydrogenase, Identification, Characteristics, *Geotrichum candidum*

## Abstract

Crude enzyme from *Geotrichum candidum* S12 exhibited high activity towards hexanol at pH 4.0, distinguishing it from currently known enzymes. To identify the dominant enzyme contributing to this activity, the crude enzyme extract was separated into different fractions by ammonium sulfate precipitation, MonoQ anion-exchange chromatography, and Sephacryl S-200 gel filtration chromatography. Afraction with high activity towards hexanol at pH 4.0 was obtained, exhibiting 38-fold improved purity and a specific activity of 3802.7 U/mg. After electrophoretic analysis, the fraction showed a molecular weight of 223 kDa by Native-PAGE and 51.4 kDa by SDS-PAGE. The purified fraction was identified as a glutamate dehydrogenase (GDH) by peptide mass fingerprinting data. This fraction showed high activity towards glutamate, α-ketoglutarate, hexanol, and isoamyl alcohol with a *K*m value of 41.74, 4.01, 20.37, and 19.37 mM, respectively, but with no activity towards methanol, ethanol, 1-propanol, and isobutanol. As a comparison, the GDH from yeast had no activity towards hexanol and other alcohols. Kinetic analysis revealed that glutamate and hexanol served as competitive inhibitors to each other for the purified GDH. The GDH showed the highest activity towards hexanol at pH 4.0 and 30 °C, and was the most stable at pH 2.2–7.0 and ≤40 °C. The presence of ADP, Fe^2+^, K^+^, and Zn^2+^ increased the enzymatic activity towards hexanol and EDTA, Pb^2+^, Mn^2+^, ATP, and DTT decreased the activity. These novel characteristics expand the reported properties of GDH and suggest the newly characterized GDH has unique potential for practical application.

**Electronic supplementary material:**

The online version of this article (doi:10.1186/s13568-016-0307-8) contains supplementary material, which is available to authorized users.

## Introduction

Hexanol and isoamyl alcohol are higher alcohols that are extensively found in foods, especially in alcoholic drinks (Styger et al. 2011). Excessive hexanol and isoamyl alcohol content distorts food flavor and taste, and even causes headaches and damage to the human neurological system (Bai et al. 2011). Ingestion of these alcohols is considered one of the main causes of alcoholic intoxication. Currently, hexanol is listed in the Hazardous Substances Data Bank (HSDB) as a hazardous substance that can inflict harm to body, with a 50% lethal dose (LD_50_) value of 720 mg/kg (Lachenmeier et al. 2008). Numerous attempts have been made to decrease hexanol and isoamyl alcohol production during food processing using absorption treatments and modified yeast strains (Callejón et al. 2009). However, few of these methods have been approved for practice due to the suppression of ethanol production or low specificity. The enzyme treatment is widely accepted in food processing due to its high specificity and gentle treatment conditions. The currently known enzymes with activity to hexanol and isoamyl alcohol are mainly alcohol acyltransferase (Yahyaoui et al. 2002) and alcohol dehydrogenase (Park et al. 2007). However, these enzymes are normally active at pH values around 7–8, and so are not suitable for wine and liquors that typically are acidic with a pH lower than 4.0. We previously found that *Geotrichum candidum* S12 demonstrated high hexanol and isoamyl alcohol activities at a pH rate around 4.0, showing a great potential to specifically reduce the level of higher alcohols in alcoholic products. The responsible enzyme was primarily identified as hexanol dehydrogenase (Zhang et al. 2013b). However, there has not been further characterization of the enzyme fraction with hexanol-degradation activity.

In food processing, *G. candidum* is used in the dairy industry, especially for products such as rind cheeses (Wouters et al. 2002). This fungus shows great potential to produce sulphur flavor compounds (Boutrou and Guéguen 2005; Spinnler et al. 2001) due to the presence of lipolytic and proteolytic activities with the lipases, proteases, amino peptidases, and transaminases, lyases, and decarboxylases (Zarevucka et al. 2005). We have limited understanding of the enzymes from *G. candidum*, especially those that have activity towards high alcohols, such as hexanol.

This work was performed using the previous study on the crude enzymes from *G. candidum* S12 as a starting point. We aimed to identify and characterize the dominant enzyme showing activity towards hexanol. Purification and amino acid sequence analysis were used for the identification study and the resulting enzyme was tested for substrate specificity, reaction and stability conditions, and metal ion influence.

## Materials and methods

### Microorganism and chemicals

*Geotrichum candidum* S12 (CCTCC AF2012005), previously isolated from soil and stored in the China Center for Type Culture Collection (Wuhan, China), was used in the study.

Methanol, ethanol, 1-propanol, n-butanol, isobutanol, hexanol, and isoamyl alcohol were purchased from Sinopharm Chemical Reagent Co. Ltd (Ningbo, China). All the other chemicals were purchased from Sigma Chemical Co. (USA).

The commercial GDH from yeast, purchased from Evergrande Parkson Biological Technology Development co. Ltd (Beijing, China), was used in the study for comparison.

### Preparation of *G. candidum* and enzyme extract

*Geotrichum candidum* S12 was cultivated aspreviously described (Zhang et al. 2013a) with some modifications. The concentration of hexanol in the medium was changed to 1.5 g/L. After cultivation, the cells were collected by centrifugation and stored at −20 °C before further study. To make an enzyme extract, 50 g of the frozen cells were ground in threefold of liquid nitrogen, and extracted with 1 L of citrate buffer (0.1 mmol/L, pH 5.8) for 30 min at 4 °C. Centrifugation at 8910×*g* for 30 min was performed to remove the cellular debris, and the supernatant was collected and used asanenzyme extract.

### Purification of putative enzyme fraction

The enzyme fraction exhibiting the highest activity towards hexanol was isolated and purified from the above prepared enzyme extract by ammonium sulfate [(NH_4_)_2_SO_4_] precipitation, MonoQ anion-exchange chromatograph, and Sephacryl S-200 gel filtration chromatography (Zhu et al. 2012). In brief, 30 and 70% saturation of (NH_4_)_2_SO_4_ was used. The precipitated protein fraction by (NH_4_)_2_SO_4_ treatment was loaded onto a MonoQ10/100 column (1.6 cm × 40 cm; GE Healthcare, Germany) using AKTA purifier TM 100, and eluted using a linear gradient program with 0–1.4 mol/L NaCl in 0.1 mmol/L citrate buffer, pH 5.8. The fractions showing activity towards hexanol were further purified via gel filtration chromatography with a Sephadex S-200 column (1.8 cm × 100 cm; GE Healthcare, Germany). The column was equilibrated with 5 volumes of 0.1 mmol/L citrate buffer, pH 5.8. Proteins were eluted at a flow rate of 1 mL/min and 1 mL fractions were collected. The fractions showing activity towards hexanol were pooled, concentrated by dialysis and lyophilization (CS110-4 Labogene, Denmark), and then the protein concentration (Bradford 1976) and enzyme activity towards hexanol were measured.

The protein fractions showing the highest activity towards hexanol were freeze-dried to powder form and stored at −20 °C. Before using, the enzyme powder was prepared in 0.1 mmol/L citrate buffer (pH 5.8) at 0.11 mg/mL with an activity of 3802 U/mg for characteristics analysis.

### HPLC and polyacrylamide gel electrophoresis analysis of the protein fraction

The HPLC measurement was carried out using a HPLC (SPD-20A, SHIMADZU Japan), on a 5 μm, 150 × 4.6 mm i.d. Wondasil-C18 column (SHIMADZU, Japan) using eluent of 0.1 mmol/L potassium phosphate buffer, pH 7.0. The purification was monitored by OD value at 280 nm based on previously reported methods (Kim et al. 1988).

Native polyacrylamide gel electrophoresis (Native-PAGE) was employed to determine the purity and relative molecular weight of the enzyme as described by Davis (1964). After electrophoresis, the protein bands on the gel were stained with coomassie brilliant blue R-250 and dehydrogenase-specific dyeing solution was implemented as described previously (Zhang et al. 2013a). In this way, the protein bands showing activity to hexanol and NADP^+^ were displayed.

Sodium dodecyl sulfate-polyacrylamide gel electrophoresis (SDS-PAGE) was also performed on the obtained enzyme fraction with the hexanol-degrading activity to determine the purity and molecular mass of the enzyme, using the method reported by Laemmli (1970), (Zhu et al. 2012).

### MALDI-TOF-MS analysisof the purified protein fraction

**Fig. 1:**
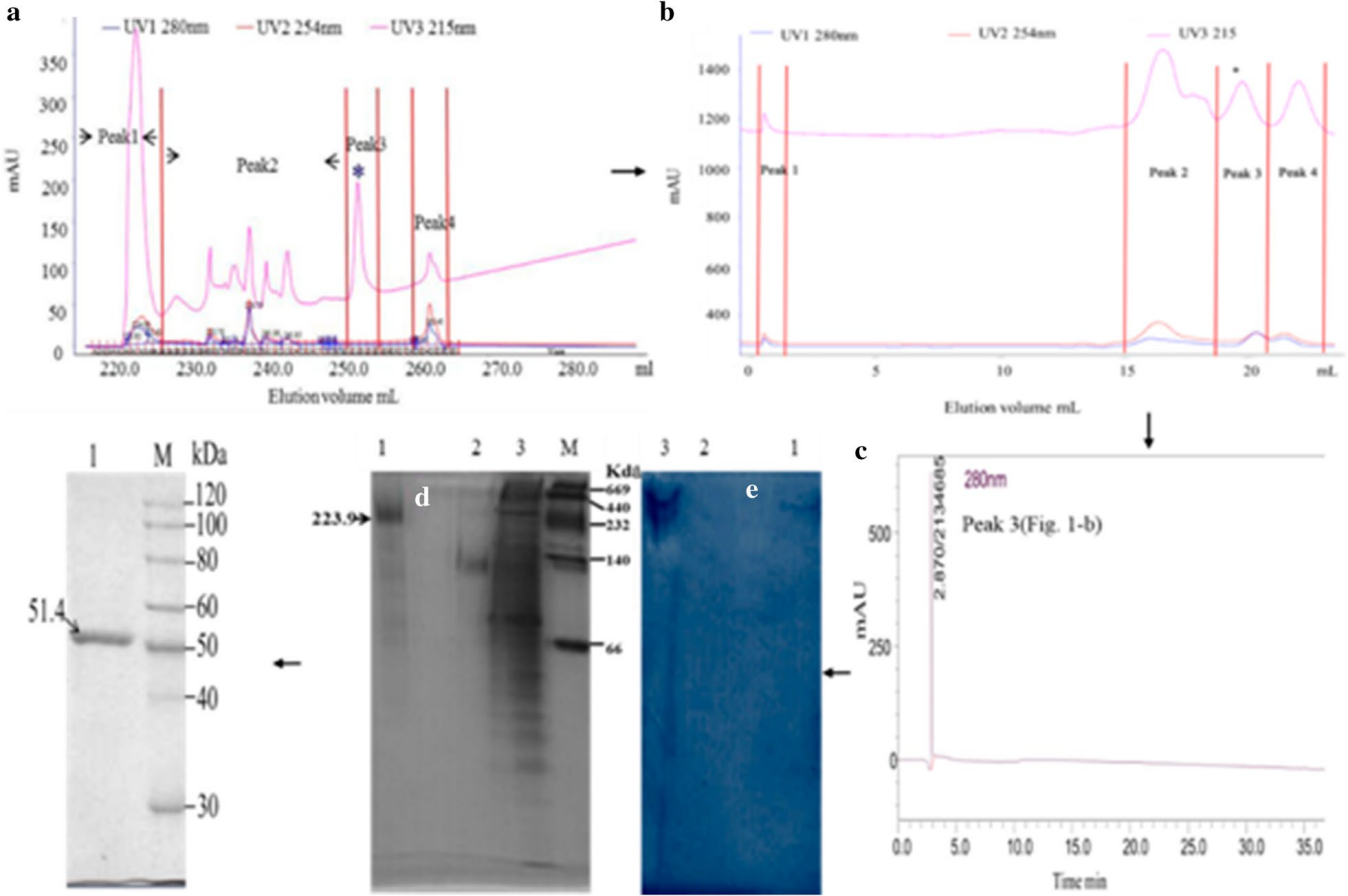
The elution profiles for the GDH extracts. *Asterisk* Indicates the fraction with a hexanol, glutamate, and α-ketoglutarate activity. The ammonium sulphate precipitated material was fractionated by ion-exchange (**a**); and the most active peaks were separated by gel filtration (**b**); the main active peak was further analyzed for purity by HPLC (**c**). The native-PAGE analysis showed the most active peak from the above steps (**d**, **e**) is lane M. *Lane M*. Native molecular mass marker, *Lane 1*. Purified GDH on gel filtration. *Lane 2*. Peak 4 from the gel filtration. *Lane 3*. Crude enzyme. The gel in (**d**) is stained with coomassie brilliant blue and the gel in (**e**) is stained with dehydrogenase active staining solution with hexanol as a substrate. The SDS-PAGE analysis shows the most active peak from the above steps (**f**): *Lane M*. Molecular mass marker, *Lane 1*. Purified GDH on Gel filtration. See “Materials and methods” for more details on the purification methods

The protein fraction with the highest hexanol-degrading activity [Fig. 1f (51.4 kDa)] was manually excised from the SDS-PAGE gel and then identified by matching using reference gels/maps and peptide mass fingerprinting using a Matrix Assisted Laser Desorption Ionization-Time of Flight mass spectrometer (MALDI-TOF MS, Applied Biosystems) (Klepsch et al. 2009). The identification of enzyme according to the peptide mass fingerprinting data was performed by MASCOT search program in the SwissProt database (Barash and Mor 1973), after excluding the trypsin autolysis products from the control spectrum.

### Assays of the enzyme activities

#### Activity towards hexanol and other higher alcohols

The enzyme activity towards hexanol and other higher alcohols was measured according to the decrease of its concentration by gas chromatography (GC). The enzyme reaction and GC analysis methods were performed as described previously (Zhu et al. 2012) except that the reaction conditions were 30 °C for 60 min. One unit (U) of the purified enzyme is the amount of enzyme required to reduce 1 μmol of hexanol per hour under these assay conditions.

#### Activity towards glutamate and α-ketoglutarate

The enzyme activity measurement was performed at 30 °C. The enzyme activity was measured by following the change in absorbance at 340 nm as described by Choudhury and Punekar (2007). One unit (U) of activity is defined as the amount of enzyme required to reduce/oxidize 1 μmol NADP^+^/NADPH/min.

### Enzymatic properties analysis

#### Substrate specificity

The substrate specificity was investigated by separately testing methanol, ethanol, 1-propanol, isobutanol, hexanol, isoamyl alcohol, glutamate, and α-ketoglutarate as substrates for the purified enzyme fraction. For each substrate, the enzyme activity was measured with different alcohol concentrations ranging from 10 to 50 mmol/L; glutamate and NH_4_^+^ concentrations-from 5 to 50 mmol/L; NADP^+^ and NADPH concentrations -from 0.01 to 0.1 mmol/L; and α-ketoglutarate concentrations—in the interval from 0.5 to 5 mmol/L. The Michaelis-Menten constant (*K*_m_) and the maximum rates of the reaction (*V*_max_) for the different substrates were determined by plotting the activity data as a function of the substrate concentration according to the method of Lineweaver and Burk (1934). The activity of the GDH from yeast towards different substrates was also determined for comparison.

#### Conditions and factor influence measurement

The influence of pH and temperature on enzyme activity and stability was tested using hexanol as the substrate. For the enzymatic conditions tests, seven levels of pH from 2.2 to 8.0 were tested for pH influence using 0.1 mol/L phosphate buffer, and different levels of temperatures from 20 to 50 °C were used to evaluate the effect of temperature on enzyme activity. For enzyme stability conditions, similar levels of pH and temperature were tested according to the residual enzyme activity after incubation in phosphate buffer at pH 4.0.

To determine the effect of metal ions on the enzyme activity, Fe^3+^, Ba^2+^, Ca^2+^, Mn^2+^, Fe^2+^, Pb^2+^, K^+^, Mg^2+^, and Zn^2+^ were added into the reaction system (pH 4.0, 30 °C) at 5, 10, or 50 mmol/L by the addition of FeCl_3_, BaCl_2_, CaSO_4_, MnCl_2_, FeSO_4_, Pb(NO_3_)_2_, KCl, MgSO_4_, or ZnSO_4_, EDTA, DTT, ATP and ADP were also added to the reaction system to test the effects of inhibitors.

## Results

### Purification of the protein fraction with activity towards hexanol

The protein fraction exhibiting activity towards hexanol was purified using a three-step procedure as shown in Fig. 1 and Table 1. After ammonium sulfate precipitation (30–70%), the protein fraction was purified 11.55-fold (Table 1). After anion-exchange, four protein fractions were obtained, peaks 1–4. Peak 2 was the lowest with activity to hexanol. The fraction eluted with saline buffer (peak 3) contained the highest enzymatic activity (2419.2 U/mg protein), and was obtained at a concentration of 1.2 mol/L NaCl (Fig. 1a). The purity of the active fraction was increased to 24.54-fold as measured by enzyme activity (Table 1). Next, the fractions, corresponding to peak3 using Sephadex S-200 column, were collected using 0.1 mmol/L citrate buffer at 4 °C (Fig. 1b). The resulted enzyme solution contained 0.011 mg/mL protein with a specific activity of 3802.7 U/mg, indicating 38.58-fold purification and 2.86% recovery of the enzymatic activity towards hexanol. The finally obtained protein fraction showed a single peak in the HPLC analysis and had a purity of 92.4% (Fig. 1c). Native-PAGE analysis indicated that the purified enzyme had a molecular weight of 223 kDa (Fig. 1d, e), and the SDS-PAGE analysis revealed a unique band of a molecular weight of 51.4 kDa, approximately one fourth of the mass of the ban don the native gel, suggesting the enzyme might contain 4 subunits (Fig. 1f).

**Table 1:** Summary of the procedure of GDH from *Geotrichum candidum*

Purification steps	Volume (mL)	Total protein (mg)	Total activity^a^ (U)	Specific activity^b^ (U mg/protein)	Recovery^c^ (%)	Purification fold^d^
Crude extract	100.0	85.11	8388.89	98.57	100.00	1
Ammonium sulfate (30–70%)	8.5	3.19	3630.43	1138.40	43.28	11.55
MonoQ anion-exchange chromatography	2.0	0.76	1838.61	2419.2	10	24.54
Sephacry S-200	5.4	0.06	239.57	3802.70	2.86	38.58

All experiments were conducted at 4 °C. The 30 and 70% ammonium sulfate fractions were pooled and then subjected to MonoQ anion-exchange chromatography

Significance of the difference (P ≤ 0.05). Mean ± SD from triplicate determinations

^a^Total activity = specific activity × total amount of protein

^b^Specific activity of GDH was tested using hexanol as a substrate

^c^Recovery = (total protein of the fraction/total activity of crude extract) × 100%

^d^Purification fold = specific activity of the fraction/specific activity of crude extract

### Identification of the obtained enzyme

**Fig. 2:**
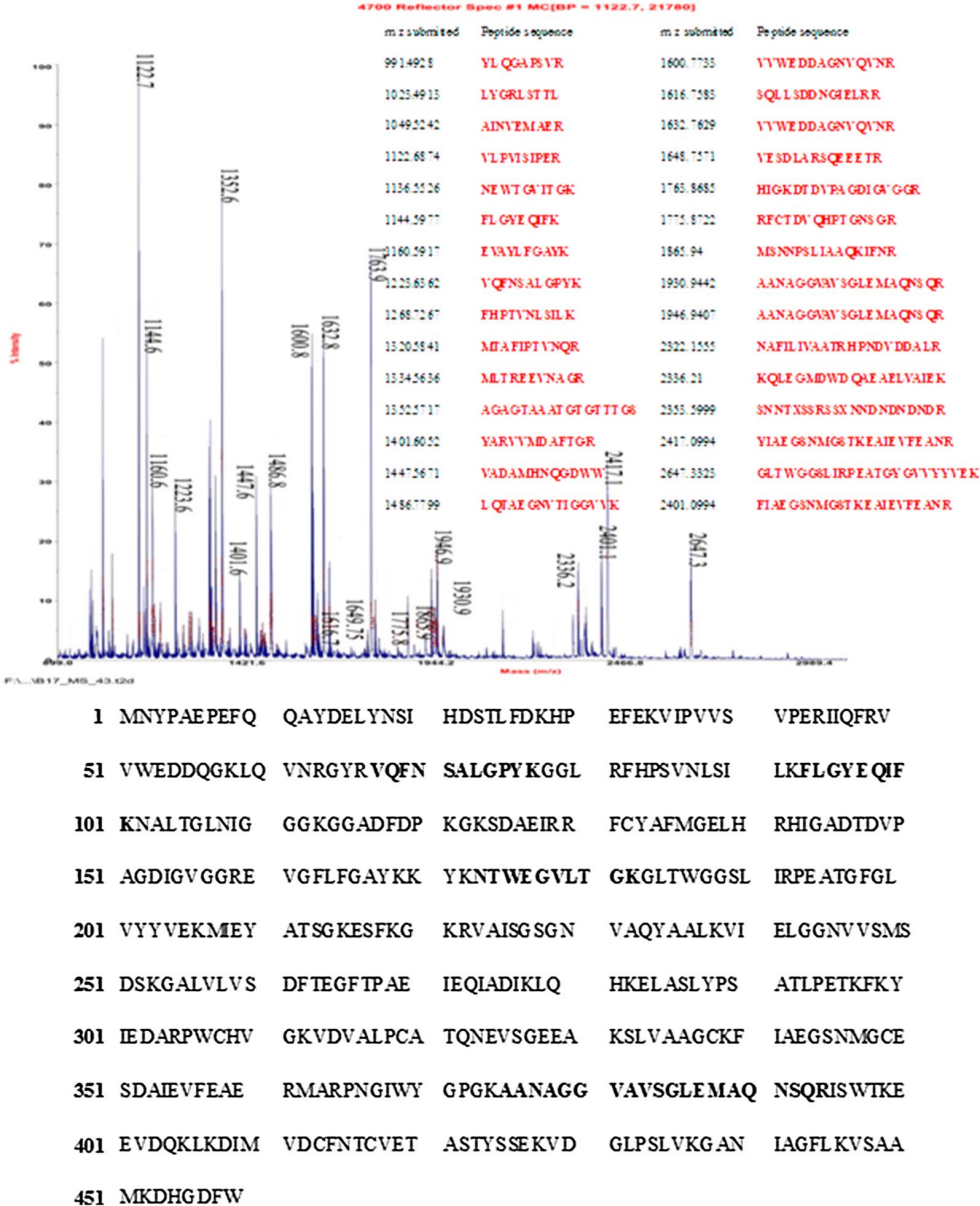
Mass spectrum obtained for tryptic peptides eluted from 1-D gel band (51.4 kDa). After a baseline correction, a background subtraction, and peak deisotoping, 30 ions were submitted to Mascot. Twenty one of the submitted ions were matched to theoretical tryptic peptides from glutamate dehydrogenase; the sequences of these peptides are shown next to the mass of the monoisotopic, singly charged ions. The full protein sequence and the sequenced peptides are in* bold*

The peptide mass fingerprinting (PMF) spectra of the subunit (molecular weight 51.4 kDa) of the targeted enzyme was acquired by MALDI-TOF MS using α-Cyano-4-hydroxycinnamic acid (CHCA) as amatrix. Figure 2 shows the MAIDI-TOF MS spectrum of the enzyme generated from the in-gel trypsin digestion, and Additional file 1: Table S1 lists the obtained PMF peaks. The MS data (Fig. 2) were subjected to searches against the non-redundant protein sequence database (MSDB) using the program Mascot (Matrix Science, London, UK; www.matrixscience.com). Matches of band (51.4 kDa) showed a similarity with m/z values of five different peptides similar to Yaliof17820p protein digested with trypsin (Additional file 1: Table S1). The m/z values of peptides were 1136.52 (NTWEGVLTGK), 1144.60 (FLGYEQIFK), 1223.64 (VQFNSALGPYK), 1903.94 (AANAGGVAVSGLEMAQNSQR), and 1946.94 (AANAGGVAVSGLEMAQNSQR), with similarity to Yaliof17820. According to the matching analysis, the purified protein was identified as the NAD(P)-binding domain of glutamate dehydrogenase, subgroup 2 (http://www.ncbi.nlm.nih.gov/Structure/cdd).

### Enzymatic properties of the purified enzyme fraction

#### Substrate specificity

**Fig. 3:**
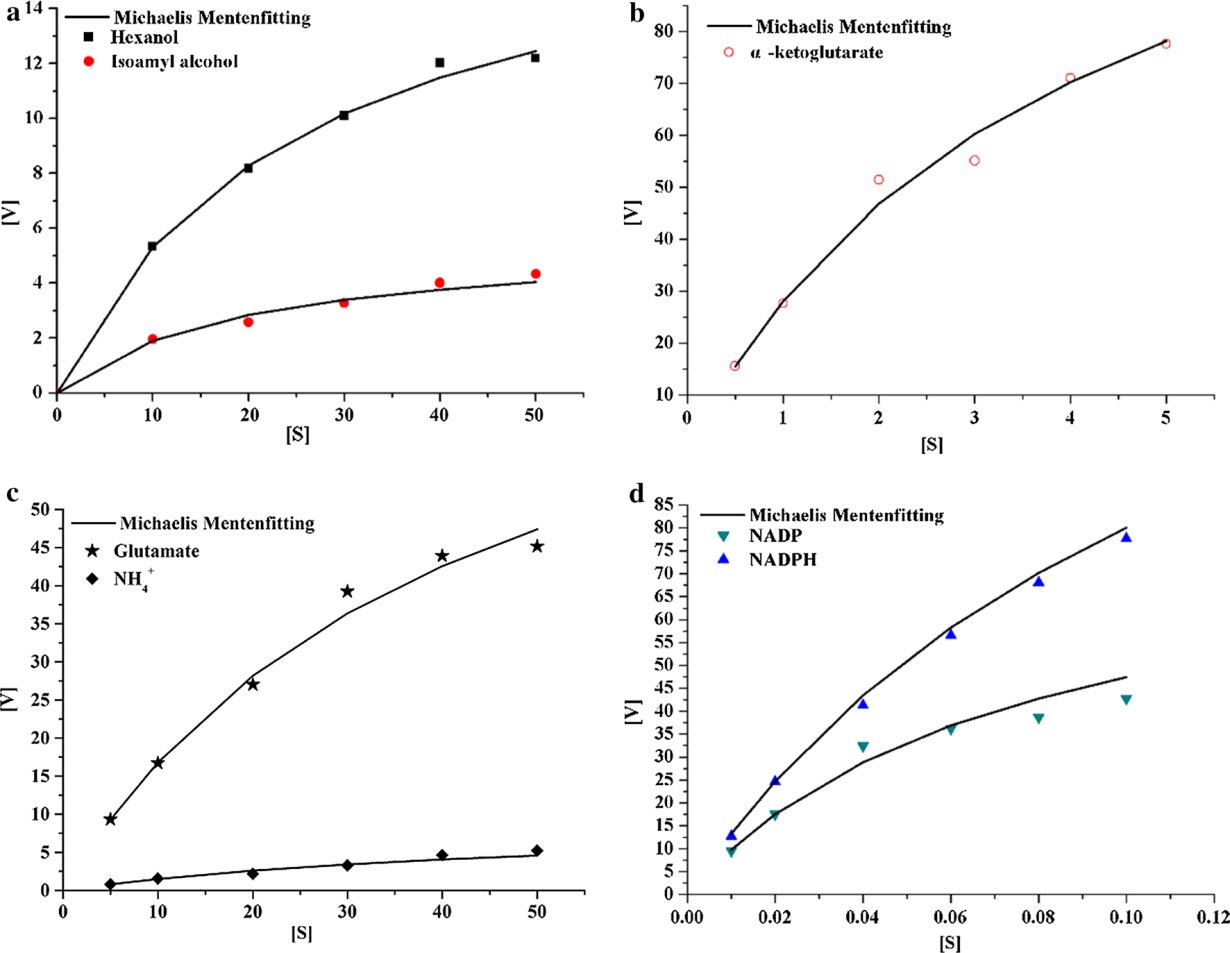
The enzyme activity for different substrates at different substrate concentrations: **a** the assay was performed at different concentrations of hexanol (*squares*) and isoamyl alcohol (*solid circles*), [S] represents the substrate concentration ranging from 10 to 50 mmol/L; **b** The assay was performed at different concentrations of α-ketoglutarate (*empty circles*) [S] represents the substrate concentration ranging from 0.5 to 5.0 mmol/L; **c** The assay was performed at different concentrations of glutamate and NH_4_^+^, [S] represents the substrate concentration ranging from 5 to 50 mmol/L; **d** The assay was performed at different concentrations of NADP^+^ and NADPH, [S] represents the substrate concentration ranging from 0.01 to 0.1 mmol/L. The conditions used in the measurement of the enzyme activity were pH 4.0 and a temperature of 30 °C

Among the tested alcohols, the purified enzyme fraction showed activity towards hexanol and isoamyl alcohol, but not towards other tested higher alcohols at all tested concentrations, from 10 to 50 mol/L. Figure 3 shows that the enzymatic reaction followed a typical first order of the Michaelis-Menten function when hexanol, isoamyl alcohol, glutamate, and α-ketoglutarate were separately tested as a substrate in the presence of NADP(H) and NH_4_^+^. As shown in Table 2, the *K*_m_ value for glutamate was tenfold of that for α-ketoglutarate. The *K*_m_ value for NADPH was nearly two-fold of that for NADP^+^. These results indicate that the enzyme has higher affinity to NADP^+^ and α-ketoglutarate than to the other substrates. Isoamyl alcohol showed lower *K*_m_ (19.37) and *V*_max_ value (5.59) than for hexanol. The characteristics of high specificity to hexanol, isoamyl alcohol, and no activity towards methanol, ethanol, 1-propanol, n-butanol, and isobutanol, indicate agreat potential application of the enzyme in food processing when the high content of hexanol and isoamyl alcohol are specifically undesirable.

**Table 2:** *K*_m_ and *V*_max_ values of GDH towards different substrates

Substrate	*K*_m_ (mol/L)	*V*_max_ [U (mg protein)^−1^]	Substrate	*K*_m_ (μmol/L)	*V*_max_ [U (mg protein)^−1^]
Methanol	–	–	Ethanol	–	–
1-propanol	–	–	NADP^+^	0.07	0.083
n-butanol	–	–	NADPH	0.13	0.182
Isobutanol	–	–	α-ketoglutarate	4.01	0.14
hexanol	20.37	16.13	Glutamate	41.74	0.87
Isoamyl alcohol	19.37	5.59	NH_4_^+^	5.35	0.003

The *K*_*m*_ and *V*_*max*_ values for each substrate were determined by measuring the initial reaction rates at various non-saturating concentrations of the substrate in the presence of a fixed volume of enzyme

–Indicates not detected

For comparison, the commercial GDH from yeast showed high specificity for glutamate and only traces of activity towards α-ketoglutarate (Table 3).

**Table 3:** Comparisons of GDH and the commercial GDH

Substrate	Ralative activity (%)^a^	
	GDH	The commercial GDH
Glutamate	100^b^	100^b^
α-ketoglutarate	136.35 ± 1.48	13 ± 0.36
Hexanol	100	ND
Isoamyl alcohol	36.73 ± 0.083	ND

^a^The enzyme activity was determined by incubating the enzyme in the presence of various compounds for 60 min at 30 °C and pH 4.0 with 9.8 mmol/L hexanol as a substrate

^b^Values represent the mean ± SD (n = 3) compared with the untreated control samples

#### Effect of pH and temperature on enzyme activity towards hexanol

**Fig. 4:**
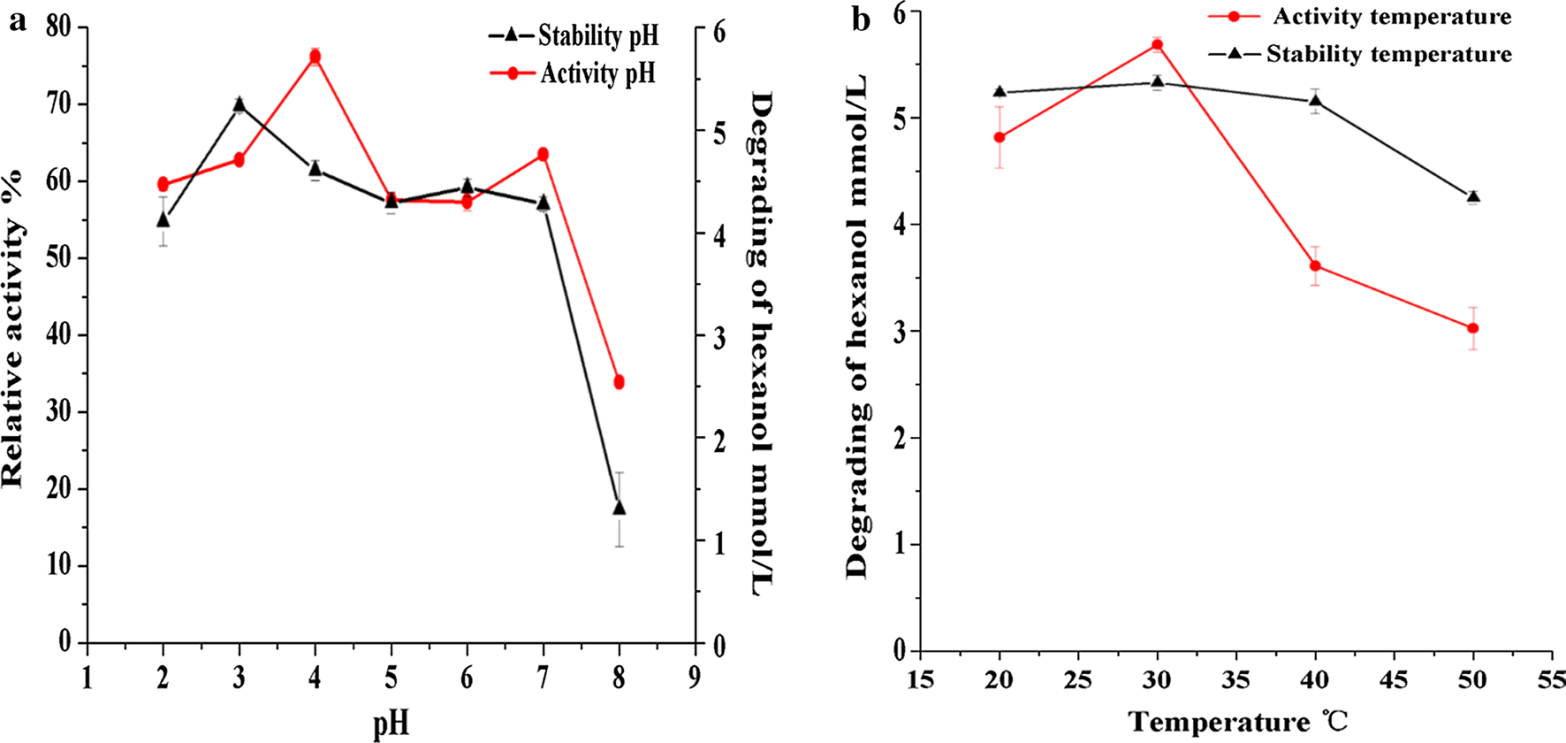
Effect of pH (**a**) and temperature (**b**) on enzyme activity and stability. In the stability tests, the residual enzyme activity was measured at 30 °C and pH 4.0 after different treatments. In the activity tests, the enzyme activity was measured at 30 °C and different pH values in the pH treatments, and at pH 4.0 and different temperatures in the temperature treatments. The bars in the curves show the standard deviations of the triplicate measurements

The enzyme activity towards hexanol was relatively stable at pH 2.2–7.0 and temperatures lower than 40 °C, but decreased sharply outside these ranges (Fig. 4). The optimum conditions for the enzyme activity were obtained at pH 4.0 and 30 °C, and outside of these boundaries the enzyme activity was significantly inhibited.

#### Effect of metal ions and inhibitors on the enzyme activity towards hexanol

Table 4 shows the results from the examination of the effects of metal ions and inhibitors, tested by the utilization of different ions or inhibitors in the reaction mixture with hexanol as a substrate. As shown, the enzyme activity increased by Fe^2+^ and K^+^ at all tested levels in a concentration-dependent manner, and by Zn^2+^ in a non-concentration-dependent manner. The metal ions K^+^ and Fe^2+^ showed the highest increase of enzyme activity at 50 mmol/L -by 93.01 and 108.35%, respectively.

**Table 4:** Effect of metal ions, chemical regents, and cofactors on the enzyme activity towards hexanol

Metal ion	Concentration (mmol/L)	Relative activity (%)^a^	Metal ion	Concentration (mmol/L)	Relative activity (%)^a^
K^+^	5	103.32 ± 3.24^b^	Ba^2+^	5	95.35 ± 2.09
	10	121.77 ± 4.53		10	97.80 ± 0.93
	50	193.01 ± 1.45		50	97.52 ± 1.55
Zn^2+^	5	106.35 ± 1.41	Mn^2+^	5	51.85 ± 4.23
	10	105.12 ± 3.56		10	42.14 ± 2.12
	50	104.23 ± 6.46		50	12.37 ± 1.98
Mg^2+^	5	67.69 ± 5.41	Pb^2+^	5	19.84 ± 0.06
	10	69.98 ± 2.47		10	6.05 ± 2.17
	50	49.75 ± 0.58		50	4.66 ± 2.66
Fe^3+^	5	95.42 ± 0.85	Ca^2+^	5	93.47 ± 1.74
	10	89.99 ± 6.01		10	50.59 ± 1.69
	50	71.50 ± 1.19		50	44.80 ± 0.96
Fe^2+^	5	102.29 ± 3.01			
	10	115.48 ± 3.40			
	50	208.35 ± 0.59			
NAD^+^	0.3	50.48 ± 1.05	EDTA	1.0	5.34 ± 0.03
NADH	0.3	5.29 ± 0.43	ATP	1.0	14.35 ± 0.84
NADP^+^	0.3	100	ADP	1.0	138.21 ± 3.72
NADPH	0.3	3.82 ± 0.78	DTT	1.0	30.72 ± 1.01

^a^The enzyme activity was determined by incubating the enzyme in the presence of various compounds for 60 min at 30 °C and pH 4.0 with 9.8 mmol/L hexanol as a substrate

^b^Values represent the mean ± SD (n = 3) compared with the untreated control samples

Metal ions Mg^2+^, Fe^3+^, Ba^2+^, Mn^2+^, Pb^2+^, and Ca^2+^ exhibited inhibition effects on the enzyme activity towards hexanol at all tested levels (5–50 mmol/L). Ba^2+^ showed an inhibitory effect at all concentrations. Other metal ions demonstrated a higher suppressing effect at higher concentrations. The heavy metal ions Pb^2+^ and Mn^2+^ showed the most significant inhibition of enzyme activity.

As expected, the addition of EDTA, ATP, and DTT led to a decrease of the enzyme activity (Table 4). When EDTA was used, only 5.34% of the enzyme activity remained. This result indicates that the enzyme is metal ion-dependent in its activity towards hexanol. The addition of ATP and DTT also decreased the enzyme activity significantly. However, the intensity of enzyme action towards hexanol was increased in the presence of ADP, indicating that this activity might be related to dehydrogenation. The inhibitory influence of denaturants (DTT) on the enzyme activity were likely caused by denaturation effects.

#### Effect of coenzymes on the enzyme activity towards hexanol

The cofactor experiments showed that the enzyme could use NADP^+^ and NAD^+^ as a hydrogen acceptor of hexanol as a substrate, but the enzyme activity reduced by 50% with the utilization of NAD^+^ as a cofactor (Table 4). The coenzymes NADH and NADPH inhibited the enzymatic activity towards hexanol to a value lower than 10%, demonstrating high enzyme specificity for catalysis of a dehydrogenation reaction.

#### Identification of the enzymatic reaction using glutamate, α-ketoglutarate, hexanol, and isoamyl alcohol as substrates

**Fig. 5:**
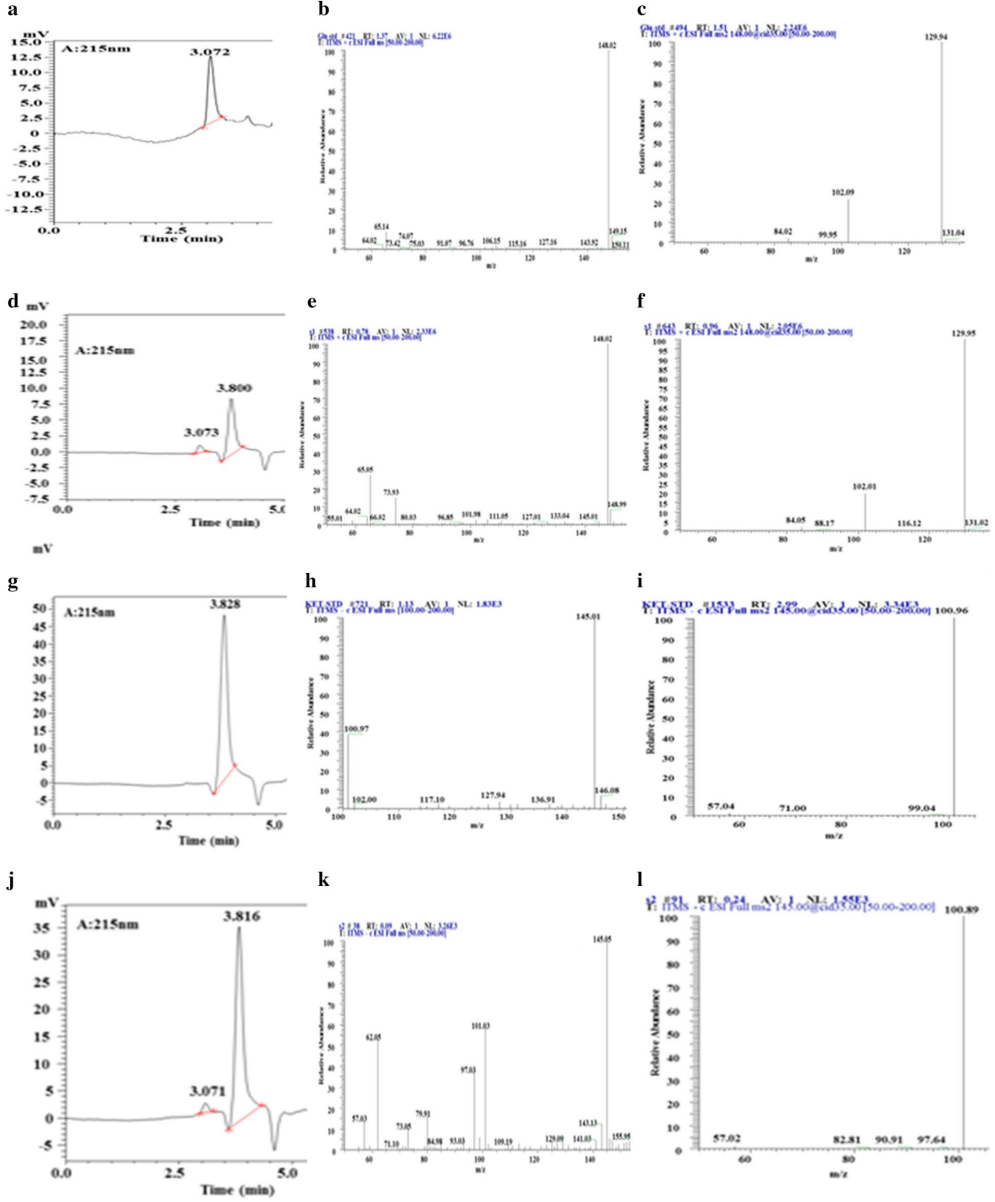
Precursor ion and daughter ion of glutamate and α-ketoglutarate standards and the samples. In the measurement, the ion reaction was set to m/z = 147.5–148.5 and m/z = 144.5–145.5, respectively; **a**, **b**, **c** are the peak time, precursor ion and daugther ion of standard glutamate, respectively; **d**, **e**, **f** are the peak time, precursor ion and daughter ion of glutamate detected in the sample, respectively; g, h, i are the peak time, precursor ion and daugther ion of standard α-ketoglutarate, respectively; **j**, **k**, **l** are the peak time, precursor ion and daugther ion of α-ketoglutarate detected in the sample

The conversion products from glutamate and α-ketoglutarate are shown in Fig. 5. As shown in the figure, the major descendant (daughter) ions of the product (retention time 3.0 min) diverted from α-ketoglutarate as a substrate with the same retention time as the glutamate standard obtained at m/z = 148.02 and m/z = 129.94. Another diverted sample (retention time 3.8 min) from glutamate as substrate showed the same retention time as α-ketoglutarate and was obtained at m/z = 145.05 and m/z = 100.80 for α-ketoglutarate, consistent with the data obtained from the corresponding standards. Therefore, glutamate and α-ketoglutarate were converted to each other by GDH from *G. candidum*, consistent with the peptide mass fingerprinting (PMF) data. Additionally, the products bioconverted by GDH from hexanol and isoamyl alcohol were identified using GC–MS (Additional file 1: Figure S1). As predicted, hexanal, and 3-methyl-butanal were detected when hexanol and isoamyl alcohol, respectively, were used as the sole substrate in the system.

We conclude that the obtained enzyme is a NADP^+^-hexanol-degrading glutamate dehydrogenase. It is novel in that it exhibits activity towards higher alcohols (hexanol and isoamyl alcohol), and posses high specificity towards glutamate and α-ketoglutarate. Our results allow predictions of the enzymatic reactions with hexanol, isoamyl alcohol, glutamate, and α-ketoglutarateas substrates.

#### Dual substrate kinetics

**Fig. 6:**
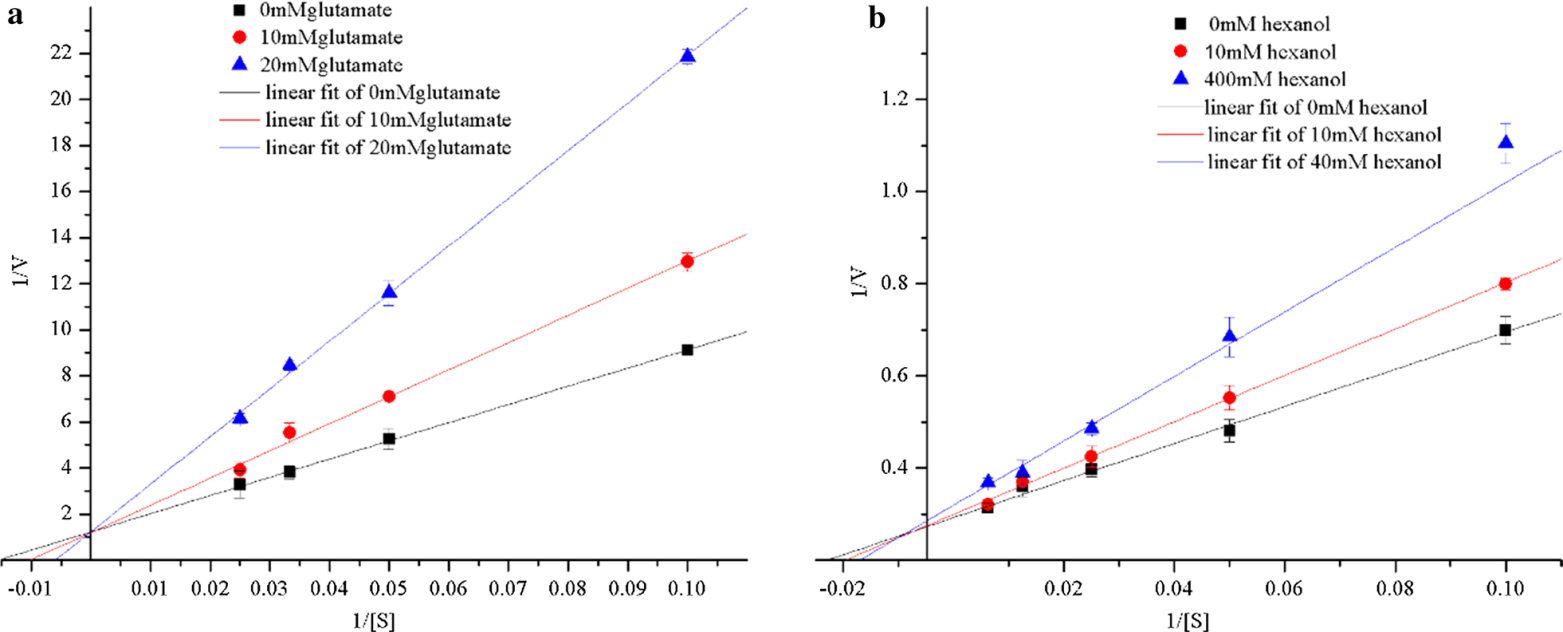
Initial velocity patterns for the obtained enzyme from *G. candidum*. **a** Hexanol was varied using the following fixed concentrations of glutamate: 0, 10 and 20 mM; **b** glutamate was varied at the following fixed concentrations of hexanol: 0,10 and 40 mM

By changing the concentrations of hexanol and glutamate, we obtained the kinetic profiles by plotting the reciprocal of the initial velocity (1/v) against the reciprocal of one substrate (1/[S]), as shown in Fig. 6. The Lineweaver-Burk double reciprocal plots of the obtained enzyme showed typical converging lines with hexanol and glutamate as the substrates at concentrations from 0 to 20 mM and 0 to 40 mM, respectively. The lines converged at the same point on the y-axis on the Lineweaver-Burk double reciprocal plot. These results indicated that hexanol and glutamate were competitive inhibitors for the purified enzyme.

In addition, the *K*m of hexanol increased by 1.49 and 2.63-fold in the presence of 10 and 20 mM glutamate, respectively. The reciprocal plots yielded Michaelis constants of the *K*m of glutamate increased by 1.25 and 2.00-fold in the presence of 10 and 40 mM hexanol, respectively. The reciprocal plots yielded Michaelis constants of 61.02 mM for glutamate and 13.43 mM for glutamate. The lower *K*m for glutamate, indicates that the enzyme has a higher affinity of the enzyme towards glutamate than to hexanol.

## Discussion

The obtained enzyme exhibits properties that distinguished it from previously reported GDH. The native-PAGE and HPLC analyses indicated that the enzyme was composed of 4 monomeric subunits. This is different from all currently reported higher alcohol-reducing enzymes that typically have subunits that have dimolecular weights of 26–80 kDa (Kataoka et al. 2006; Kulig et al. 2013; Yamada-Onodera et al. 2007). By examination of the phylogenetic tree of the NAD(P)-dependent ADH family of proteins and related amino acid sequences in the existing protein database (NCBI) (Jeon et al. 2008), the *G. candidum* S12 GDH obtained in this study may exhibit alcohol dehydrogenase and GDH (EC1.4.1.3) activity for activity towards glutamate and higher alcohols, respectively. The unique characteristics of the obtained GDH with optimum pH at 4.0 and NAD inhibition distinguish it from other GDHs that were not been previously reported to have activity towards higher alcohols. The reported NADP^+^-dependent GDH from *Kluyveromyces marxianus* showed a significantly lower specific growth rate on ethanol, and its activity was identical with the presence of glucose (Morais-Júnior 2003). Furthermore, the obtained enzyme was also different from all reported NAD- and NADP-dependent GDHs that occupy a key position in inter-linking carbon and nitrogen metabolism in fungi. In *Benjaminella poitrasii*, Aradhana et al. (Amin et al. 2004) observed a morphology-associated increase of NADP-dependent GDH during the yeast-mycelium transition of a dimorphic fungus, and Joshi et al. (2010) suggested that a low ratio between NADP-GDH/NAD-GDH may play a role in the yeast-transition switch from aerobic to fermentation metabolism shunting pyruvate to acetaldehyde. GDH catalyzes the reversible reductive amination of α-ketoglutarate to produce glutamate (Díaz et al. 2006; Ferrer et al. 1996; Martínez-Espinosa et al. 2006; Pire et al. 2014). Most studies have focused on nitrogen production, ignoring the carbon sources even induction or repression of GDH (Barash and Mor 1973; Liu et al. 2012). This is the first report of a GDH originating from *G. candidum* with activity towards higher alcohol and high activity at pH 4.0. This enzyme may prove useful for higher alcohol content control in food industry.

The novel characteristics of the obtained enzyme distinguish it from all reported alcohol dehydrogenases (ADH). First, almost all of the previously described ADHs showed activity towards ethanol, and some of them additionally showed activity towards n-butanol, 1-pentanol, and hexanol (Mackintosh and Fewson 1988). Second, most known ADH and GDH showed optimum activity inneutraland slightly alkaline conditions (Carrigan et al. 2005). Third, the reported NAD-dependent ADH from *G. capitatum* had the most favorable conditions of pH 4.5–5.5 and 40–50 °C for optimal activity towards 2-propanol, 2-butanol, 2-pentanol, 2-hexanol, but without activity towards hexanol and glutamate (Yahyaoui et al. 2002). Optimum temperatures of 35 and 30 °C were reported for a NAD-dependent ADH from *Pseudomonas frederiksbergensis* (Jollivet et al. 1994) and *Rhodococcus* sp.GK1 (Daigle et al. 1999), respectively, and NADPH-dependent ADH from *Ralstonia* sp. (Davis 1964), when ethanol was used as the substrate. Unlike all of these, the enzyme obtained in this study did not show activity towards ethanol and methanol but showed the highest activity towards hexanol at pH 4.0. Also, the obtained enzyme was different from alcohol oxidases because this is the first report of effects of NAD and NADH on alcohol oxidase activity towards hexanol. Therefore, the obtained enzyme in this study is unique from previously reported GDH and ADH.

In addition, the low optimum pH of the obtained enzyme indicates it has great potential for application in food processing, especially in alcoholic drinks. In addition, *G. candidum*is safe for food processing, as it is widely utilized in cheese ripening (Jollivet et al. 1994). The presence of this fungus and its products is widespread in foods, especially in cheese, and is positively appreciated by consumers (Daigle et al. 1999). Some strains of *Geotrichum* genus produce esters that exhibit some specific fruit aromas (Defilippi et al. 2009). Therefore, *G. candidum* S12 may have a great potential for use in food processing, particularly the acidic environment of wine and alcoholic beverages (pH 2.7–3.8).

The increase of activity towards hexanol by the addition of Zn^2+^ was consistent with previous reports that most alcohol dehydrogenases, including alcohol dehydrogenase, contain zinc (Brandt et al. 2009). Here, Zn ion plays a structural role and is crucial to enzyme stability. In addition, a large group of ADHs are metal ion-deactivated, predominantly by reacting with the -SH residues of the enzymatic amino acid structure (Leskovac et al. 2002). These ions may also interact with the free carboxyl groups of the enzyme, thus altering the enzyme’s conformation, which could partially deactivate the enzyme. In this study, Ca^2+^ inhibited enzyme activity at all concentrations (10–50 mmol/L), but Fe^2+^, K^+^, and Zn^2+^ increased activity at different optimal concentrations. This was consistent with the effects of Fe^2+^ on the alcohol dehydrogenase from *Candida* sp. (Yabe et al. 1992) and the effect of K^+^ on the enzymes from *Thermus thermophiles* (Pennacchio et al. 2008) and *Sulfolobus solfataricus* (Yabe et al. 1992).

Other compounds affecting enzyme activities are chelates (e.g. EDTA), which influence the activation of the enzyme by blocking a metal (usually zinc) and interacting in a central place at the active site of the enzyme. In this study, the enzyme activity towards hexanol was reduced almost completely in the presence of EDTA, possibly due to the action of EDTA to remove necessary metal ions from the enzyme.

Another novel property of the obtained enzyme is that it exhibited substrate kinetics towards higher alcohols and glutamate. There are two modes of reaction mechanism for bisubstrate enzymes, sequential and ping pong. These two mechanisms can be distinguished by plotting the double reciprocal plots that either converge at any point or are parallel to each other, depicting the sequential or ping-pong reaction mechanism, respectively (Garrett 2008). The double reciprocal plots of velocity versus hexanol and glutamate concentrations in this study were straight lines crossing each other at a certain point, indicating that the obtained enzyme followed a sequential mode of reaction mechanism. In previous studies, many NAD^+^-dependent ALDHs showed a sequential reaction mechanism (Alam et al. 2016; Henehan and Tipton 1992).

## Additional file

**Additional file 1.** Additional figure and table.

## Abbreviations

GDH: glutamate dehydrogenase
ADH: alcohol dehydrogenase
ADP: adenosine diphosphate
ATP: adenosine triphosphoric acid
EDTA: ethylene diamine tetraacetic acid
DTT: disulfide generation sue sugar alcohols
HSDB: Hazardous Substances Data Bank
LD50: 50% lethal dose
HPLC: high performance liquid chromatography
Native-PAGE: native polyacrylamide gel electrophoresis
SDS-PAGE: sodium dodecyl sulfate-polyacrylamide gel electrophoresis
NADP: nicotinamide adenine dinucleotide phosphate
MALDI-TOF MS: matrix assisted laser desorption ionization-time of flight mass spectrometer
GC: gas chromatography
*K*_m_: Michaelis-Menten constant
*V*_max_: maximum rates of the reaction
PMF: peptide mass fingerprinting
CHCA: α-cyano-4-hydroxycinnamic acid

## References

[CR1] Alam MF, Laskar AA, Choudhary HH, Younus H (2016). Human salivary aldehyde dehydrogenase: purification, kinetic characterization and effect of ethanol, hydrogen peroxide and sodium dodecyl sulfate on the activity of the enzyme. Cell Biochem Biophys.

[CR2] Amin A, Joshi M, Deshpande MV (2004). Morphology-associated expression of NADP-dependent glutamate dehydrogenases during yeast-mycelium transition of a dimorphic fungus *Benjaminiella poitrasii*. Antonie Van Leeuwenhoek.

[CR3] Bai J, Baldwin EA, Imahori Y, Kostenyuk I, Burns J, Brecht JK (2011). Chilling and heating may regulate C6 volatile aroma production by different mechanisms in tomato (*Solanum lycopersicum*) fruit. Postharvest Biol Technol.

[CR4] Barash I, Mor H (1973). Regulation of nicotinamide adenine dinucleotide phosphate-specific glutamate dehydrogenase in germinated spores of *Geotrichum candidum*. Plant Physiol.

[CR5] Boutrou R, Guéguen M (2005). Interests in *Geotrichum candidum* for cheese technology. Int J Food Microbiol.

[CR6] Bradford MM (1976). A rapid and sensitive method for the quantitation of microgram quantities of protein utilizing the principle of protein-dye binding. Anal Biochem.

[CR7] Brandt EG, Hellgren M, Brinck T, Bergman T, Edholm O (2009). Molecular dynamics study of zinc binding to cysteines in a peptide mimic of the alcohol dehydrogenase structural zinc site. Phys Chem Chem Phys.

[CR8] Callejón RM, Tesfaye W, Torija MJ, Mas A, Troncoso AM, Morales ML (2009). Volatile compounds in red wine vinegars obtained by submerged and surface acetification in different woods. Food Chem.

[CR9] Carrigan JB, Coughlan S, Engel PC (2005). Properties of the thermostable glutamate dehydrogenase of the mesophilic anaerobe *Peptostreptoccus asaccharolyticus* purified by a novel method after over-expression in an *Escherichia coli* host. FEMS Microbiol Lett.

[CR10] Choudhury R, Punekar NS (2007). Competitive inhibition of glutamate dehydrogenase reaction. FEBS Lett.

[CR11] Daigle P, Gélinas P, Leblanc D, Morin A (1999). Production of aroma compounds by *Geotrichum candidum* on waste bread crumb. Food Microbiol.

[CR12] Davis BJ (1964). Disc electrophoresis—II method and application to human serum proteins. Ann N Y Acad Sci.

[CR13] Defilippi BG, Manríquez D, Luengwilai K, González-Agüero M (2009). Aroma volatiles: biosynthesis and mechanisms of modulation during fruit ripening. Adv Bot Res.

[CR14] Díaz S, Pérez-Pomares F, Pire C, Ferrer J, Bonete MJ (2006). Gene cloning, heterologous overexpression and optimized refolding of the NAD-glutamate dehydrogenase from *Haloferax mediterranei*. Extremophiles.

[CR15] Ferrer J, Pe´Rez-Pomares F, Bonete MJ (1996). NADP-glutamate dehydrogenase from the halophilic archaeon Haloferax mediterraner enzyme purification, N-terminal sequence and stability. FEMS Microbiol Lett.

[CR16] Garrett JM (2008). Amino acid transport through the *Saccharomyces cerevisiae* Gap1 permease is controlled by the Ras/cAMP pathway. Int J Biochem Cell Biol.

[CR17] Henehan GT, Tipton KF (1992). Steady-state kinetic analysis of aldehyde dehydrogenase from human erythrocytes. Biochem J.

[CR18] Jeon YJ, Fong JCN, Riyanti EI, Neilan BA, Rogers PL, Svenson CJ (2008). Heterologous expression of the alcohol dehydrogenase (adhI) gene from *Geobacillus thermoglucosidasius* strain M10EXG. J Biotechnol.

[CR19] Jollivet N, Chataud J, Vayssier Y (1994). Production of volatile compounds in model milk and cheese media by eight strains of *Geotrichum candidum* Link. J Dairy Res.

[CR20] Joshi CV, Ghormade V, Kunde P, Kulkarni P, Mamgain H, Bhat S, Paknikar KM, Deshpande MV (2010). Flocculation of dimorphic yeast *Benjaminiella poitrasii* is altered by modulation of NAD-glutamate dehydrogenase. Bioresour Technol.

[CR21] Kataoka M, Nakamura Y, Urano N, Ishige T, Shi G, Kita S, Sakamoto K, Shimizu S (2006). A novel NADP^+^ -dependent l -1-amino-2-propanol dehydrogenase from *Rhodococcus erythropolis* MAK154: a promising enzyme for the production of double chiral aminoalcohols. Lett Appl Microbiol.

[CR22] Kim K, Kim IH, Lee KY, Rhee SG, Stadtman ER (1988). The isolation and purification of a specific “protector” protein which inhibits enzyme inactivation by a thiol/Fe(III)/O2 mixed-function oxidation system. J Biol Chem.

[CR23] Klepsch M, Schlegel S, Wickström D, Friso G, Wijk KJV, Persson JO, Gier JWD, Wagner S (2009). Immobilization of the first dimension in 2D blue native/SDS–PAGE allows the relative quantification of membrane proteomes. Methods.

[CR24] Kulig J, Frese A, Kroutil W, Pohl M, Rother D (2013). Biochemical characterization of an alcohol dehydrogenase from *Ralstonia* sp.. Biotechnol Bioeng.

[CR25] Lachenmeier DW, Haupt S, Schulz K (2008). Defining maximum levels of higher alcohols in alcoholic beverages and surrogate alcohol products. Regul Toxicol Pharmacol.

[CR26] Laemmli UK (1970). Cleavage of structural proteins during the assembly of the head of bacteriophage T4. Nature.

[CR27] Leskovac V, Trivić S, Pericin D (2002). The three zinc-containing alcohol dehydrogenases from baker’s yeast, *Saccharomyces cerevisiae*. FEMS Yeast Res.

[CR28] Lineweaver H, Burk D (1934). The determination of enzyme dissociation constant. J Am Chem Soc.

[CR29] Liu Z, Zhou Y, Liu S, Zhong H, Zhang C, Kang X, Liu Y (2012). Characterization and dietary regulation of glutamate dehydrogenase in different ploidy fishes. Amino Acids.

[CR30] Mackintosh RW, Fewson CA (1988). Benzyl alcohol dehydrogenase and benzaldehyde dehydrogenase II from *Acinetobacter calcoaceticus*. Purification and preliminary characterization. Biochem J.

[CR31] Martínez-Espinosa RM, Esclapez J, Bautista V, Bonete MJ (2006). An octameric prokaryotic glutamine synthetase from the haloarchaeon Haloferax mediterranei. FEMS Microbiol Lett.

[CR32] Morais-Júnior MAD (2003). The NADP+ -dependent glutamate dehydrogenase of the yeast *Kluyveromyces marxianus* responds to nitrogen repression similarly to *Saccharomyces cerevisiae*. Braz J Microbiol.

[CR33] Park YC, San KY, Bennett GN (2007). Characterization of alcohol dehydrogenase 1 and 3 from *Neurospora crassa* FGSC2489. Appl Microbiol Biotechnol.

[CR34] Pennacchio A, Pucci B, Secundo F, La CF, Rossi M, Raia CA (2008). Purification and characterization of a novel recombinant highly enantioselective short-chain NAD(H)-dependent alcohol dehydrogenase from *Thermus thermophilus*. Appl Environ Microbiol.

[CR35] Pire C, Martínez-Espinosa RM, Pérez-Pomares F, Esclapez J, Bonete MJ (2014). Ferredoxin-dependent glutamate synthase: involvement in ammonium assimilation in Haloferax mediterranei. Extremophiles.

[CR36] Spinnler HE, Berger C, Lapadatescu C, Bonnarme P (2001). Production of sulfur compounds by several yeasts of technological interest for cheese ripening. Int Dairy J.

[CR37] Styger G, Dan J, Bauer FF (2011). Identifying genes that impact on aroma profiles produced by *Saccharomyces cerevisiae* and the production of higher alcohols. Appl Microbiol Biotechnol.

[CR38] Wouters JTM, Ayad EHE, Hugenholtz J, Smit G (2002). Microbes from raw milk for fermented dairy products. Int Dairy J.

[CR39] Yabe M, Shitara K, Kawashima J, Shinoyama H, Ando A, Fujii T (1992). Purification and properties of an alcohol dehydrogenase isozyme from a methanol-using yeast, *Candida* sp. N-16. Biosci Biotechnol Biochem.

[CR40] Yahyaoui FEL, Wongs-Aree C, Latché A, Hackett R, Grierson D, Pech JC (2002). Molecular and biochemical characteristics of a gene encoding an alcohol acyl-transferase involved in the generation of aroma volatile esters during melon ripening. Eur J Biochem.

[CR41] Yamada-Onodera K, Fukui M, Tani Y (2007). Purification and characterization of alcohol dehydrogenase reducing N -benzyl-3-pyrrolidinone from *Geotrichum capitatum*. J Biosci Bioeng.

[CR42] Zarevucka M, Saman D, Wimmer Z, Kejfk Z, Demnerova K (2005). Enantioselective properties of induced lipases from *Geotrichum*. Enzyme Microb Technol.

[CR43] Zhang J, Shi J, Liu Y (2013). Substrates and enzyme activities related to biotransformation of resveratrol from phenylalanine by *Alternaria* sp. MG1. Appl Microbiol Biotechnol.

[CR44] Zhang J, Shi J, Lv H, Liu Y (2013). Induction of hexanol dehydrogenase in *Geotrichum* spp. by the addition of hexanol. Appl Microbiol Biotechnol.

[CR45] Zhu J, Shi J, Pan Z (2012). Purification and characterization of a hexanol-degrading enzyme extracted from apple. J Agric Food Chem.

