# Supplementary material for: Purification and characterization of a novel glutamate dehydrogenase from Geotrichum candidum with higher alcohol and amino acid activity
Source: AMB Express. 2017 Jan 3;7:9. doi: 10.1186/s13568-016-0307-8 (PMC5209314; doi:10.1186/s13568-016-0307-8)
Supplement: Supplementary file 1 — Additional file 1. Additional figure and table. [file 13568_2016_307_MOESM1_ESM.docx]

AMB Express

**Purification and characterization of a novel glutamate dehydrogenase from *Geotrichum candidum* with higher alcohol and amino acid activity**

Jing Zhu^a,b#^, XiaoguangXu^a#^, Kuan Lu^a^, Xinglong Wang^c^, DongyanShao^a^, Junling Shi^a*^,HuiYang^a^, QingshengHuang^a^

^a^Key Laboratory for Space Bioscience and Biotechnology, School of Life Sciences, Northwestern Polytechnical University, 127 Youyi West Road, Xi’an, Shaanxi Province 710072, China

^b^Department of Food Science, Xinyang College of Agriculture and Forestry, New 24 street of yangshan new district, Xinyang, Henan Province 464000, China

^c^College of Enology, Northwest A&F University, 23 Xinong Road, Yangling, Shaanxi Province 712100, China

*Corresponding Author. Shi JL, E-mail: sjlshi2004@nwpu.edu.cn; Tel: +86-029-88460543; Fax: +86-029-88460543. #These authors contributed equally to this work.

Fig. S1. Total ion chromatograms from GC-MS analysis of the products resulting from the reactions of the obtained enzyme with different higher alcohols.


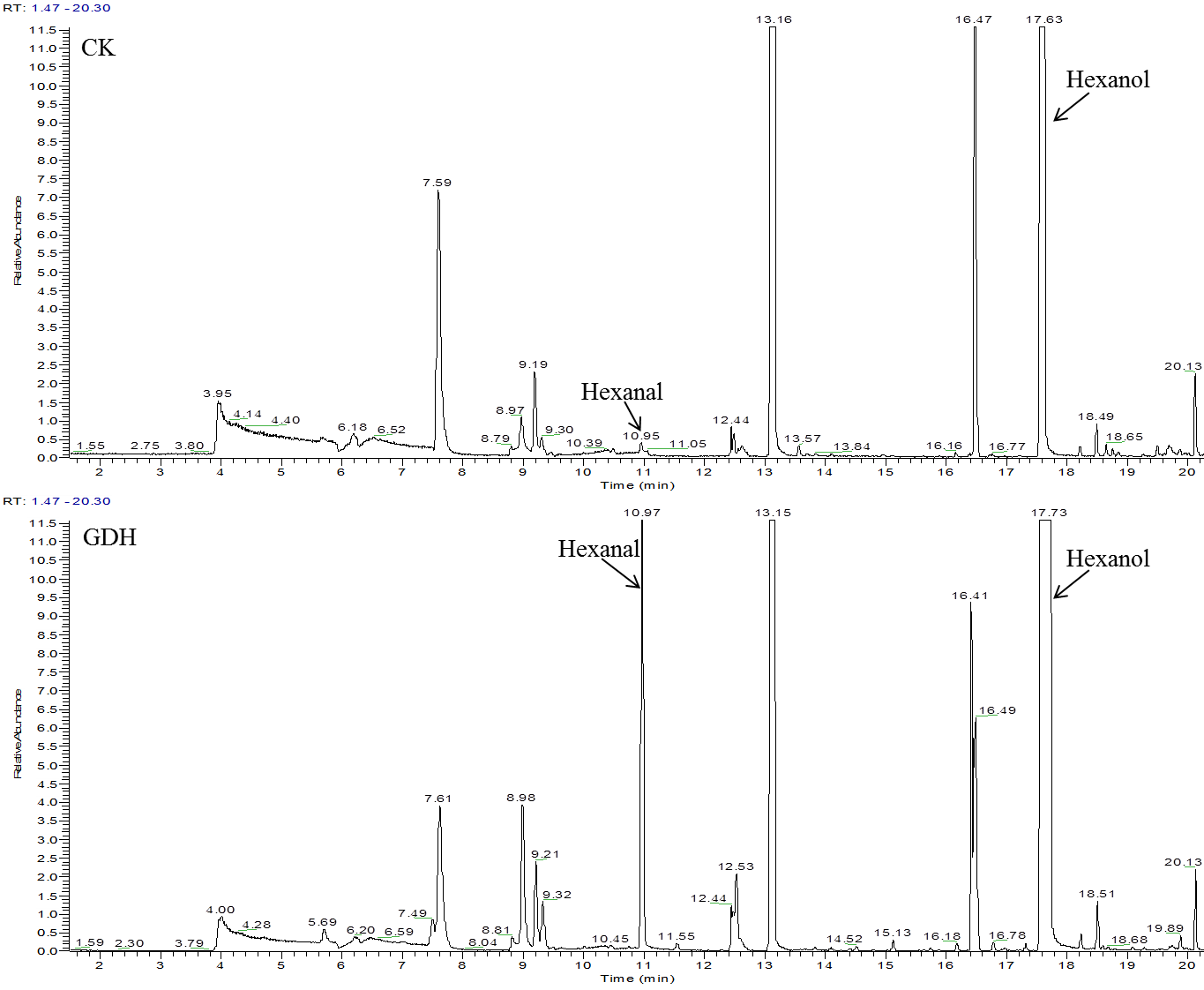


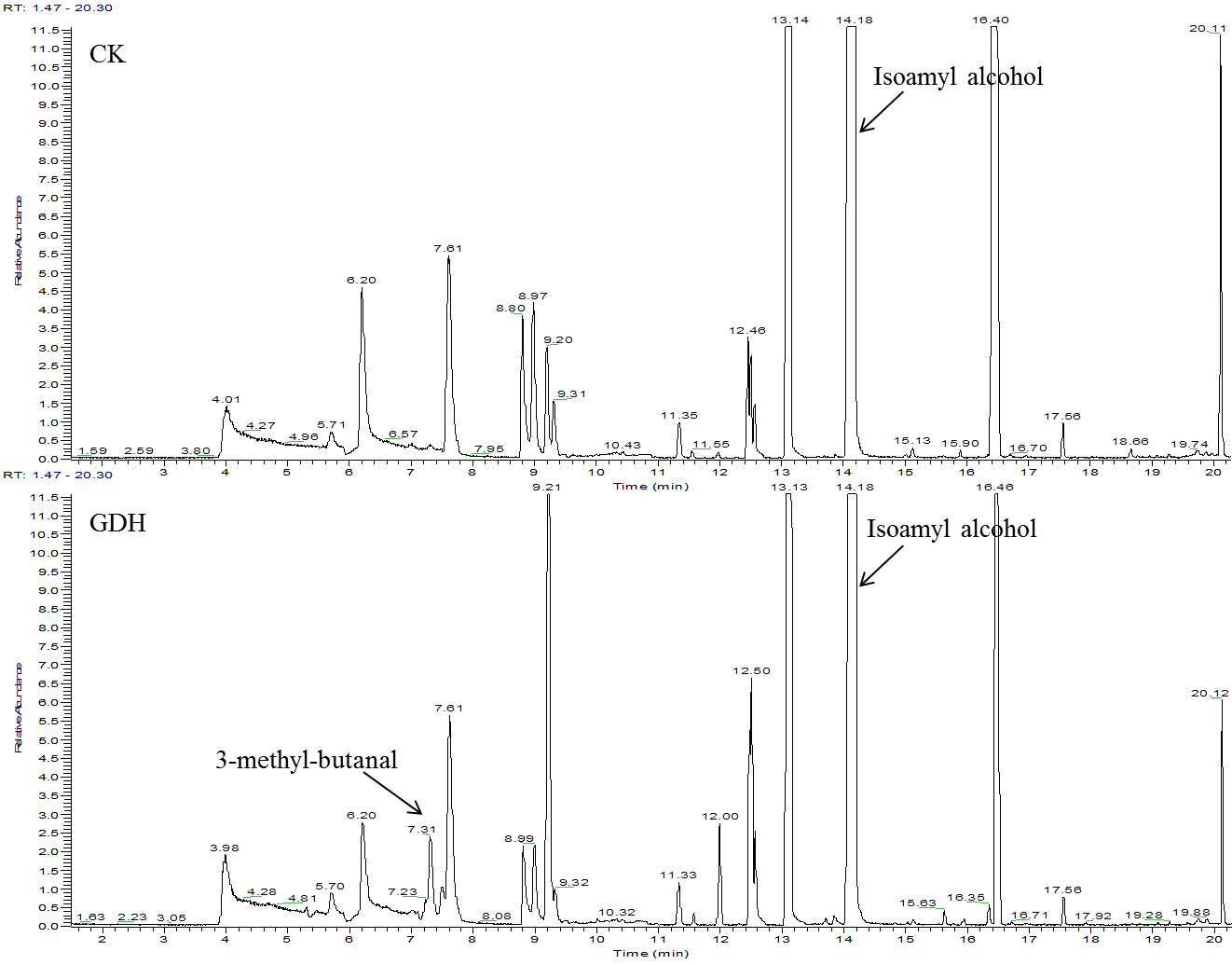


Fig. S1

Table S1 Data on identification of targeted protein by peptide sequencing (Fig. 2)*^a^*

| Identified protein | Origin | No. of peptides | p*I* | Coverage (%) | Genbank accession | Mr |
| --- | --- | --- | --- | --- | --- | --- |
| Unnamed protein product | Kuraishia capsulate CBS 1993 | 6(4) | 5.54 | 12% | gi\|549050515 | 49.45 |
| NADP-specific glutamate dehydrogenase | Sclerotinia borealis F-4157 | 4(4) | 5.93 | 11% | gi\|563289043 | 49.17 |
| Glutamate dehydrogenase | *Flammulina velutipes* | 4(4) | 6.20 | 10% | gi\|523788649 | 49.30 |
| Yaliof17820p | *Yarrowia lipolytical* | 5(4) | 5.48 | 10% | gi\|50556290 | 50.27 |

^a^Note: Mascot searches were performed against the NCB Inr database.
